# Supplementary material for: HIVprotI: an integrated web based platform for prediction and design of HIV proteins inhibitors
Source: J Cheminform. 2018 Mar 9;10:12. doi: 10.1186/s13321-018-0266-y (PMC5845081; doi:10.1186/s13321-018-0266-y)
Supplement: Supplementary file 2 — Additional file 2. Source code of HIVProtI web server. [file 13321_2018_266_MOESM2_ESM.zip › HIVprotI_Source-code/hivprotI/jsmol/testPC.htm]

Test of NCBI/PubChem and NCI/CADD AJAX calls


| NCBI/PubChem | NCI/CIR |
| --- | --- |
| test http sync (failing in FF) test https sync (works)   test http async (failing in FF) test https async (works)     My observations are that PubChem access is now  (as of 3/7/2014) by https only. This is a problem. | test http sync (works) test https sync (will time out)   test http async (works) test https async (will time out)     NCI's Chemical Identifier Resolver does not provide https service.  This is a different problem. |
